# Supplementary material for: Deep quantification of substrate turnover defines protease subsite cooperativity
Source: Mol Syst Biol. 2024 Oct 28;20(12):3. doi: 10.1038/s44320-024-00071-4 (PMC11612144; doi:10.1038/s44320-024-00071-4)
Supplement: Supplementary file 11 — Expanded View Figures [file 44320_2024_71_MOESM11_ESM.pdf]

Expanded View Figures

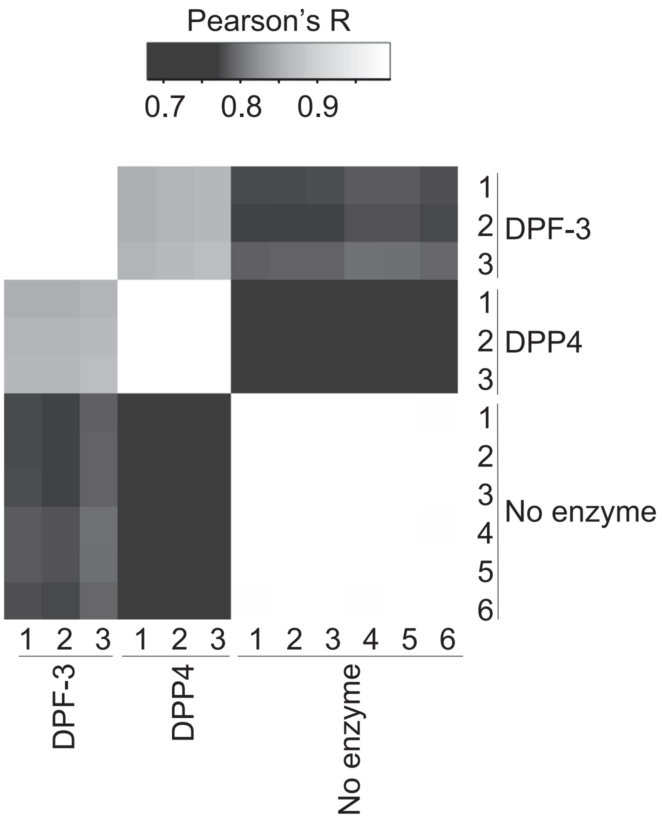

Figure EV1. Determination of DPP4 cleavage motif.

Pairwise correlation plot of identified peptides for each experimental condition and sample as indicated, showing Pearson's correlation coefficient. Label numbers indicate technical replicates. In subsequent quantitative analyses, three "No enzyme" control replicates were used for comparison to DPF-3-digested samples, the other three for comparison to DPP4-digested samples.

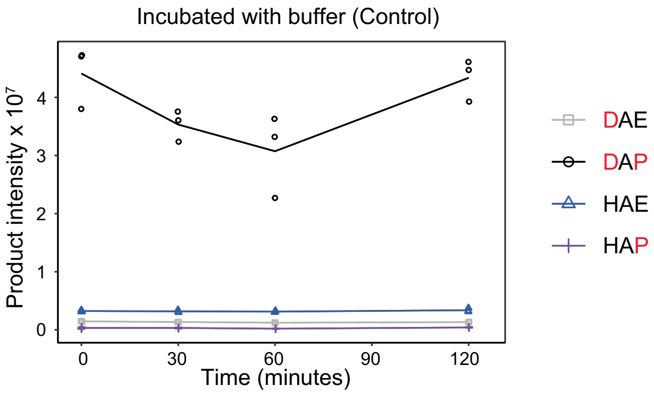

Figure EV2. Products are detectable in the synthetic peptide cleavage assay.

Zoom-in on the panel from Fig. 4B showing product peptide intensities over time following incubation of a GLP-$1_{(7\text{-}36)}$-derived peptide and its variants in buffer only (negative control). Time-invariant trace levels (<1% of signal detected in the presence of enzyme) of the expected product peptides are detected as contaminants of the peptide substrates, confirming that all hypothetical products are detectable in the assay.

 © The Author(s)

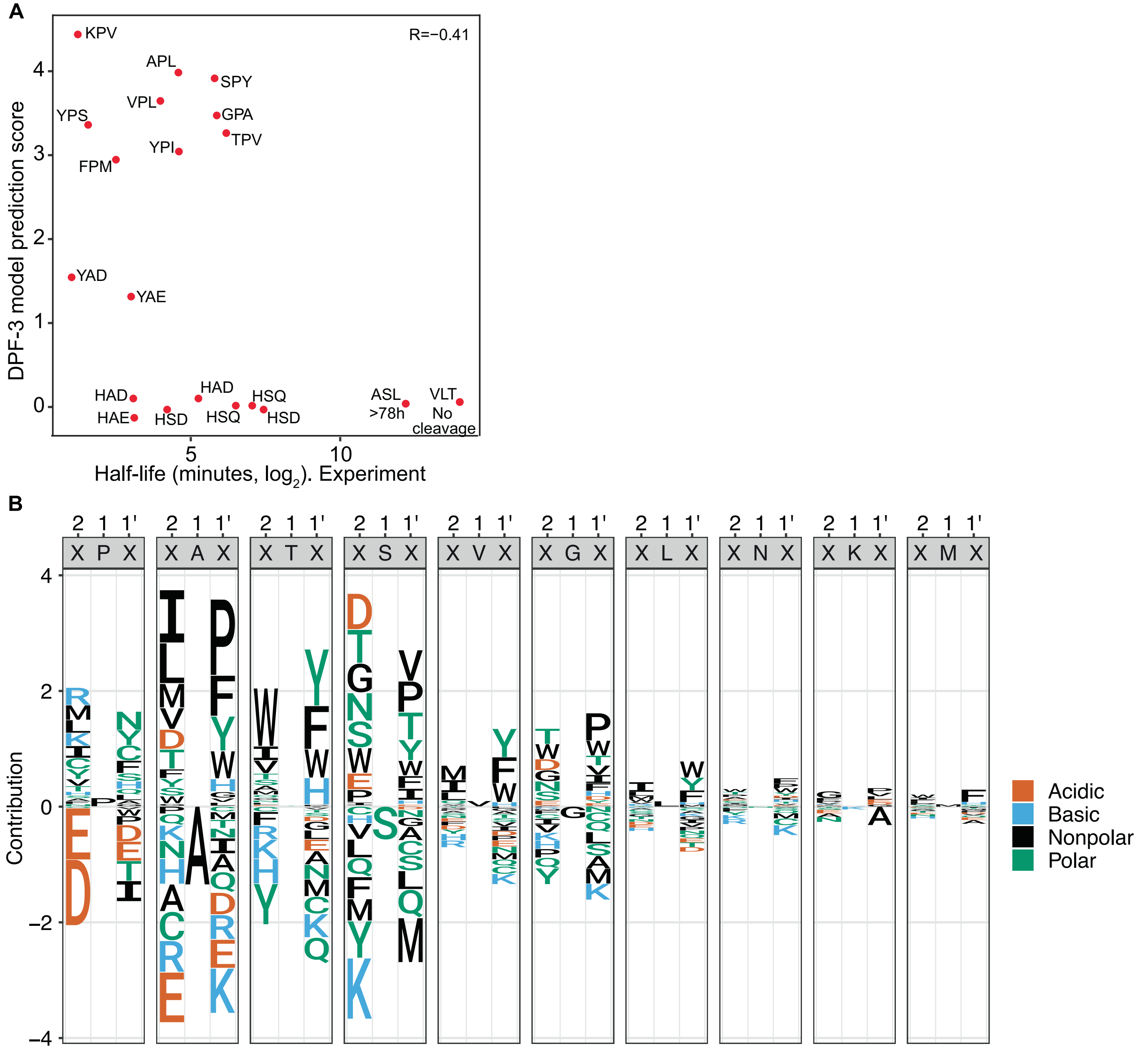

Figure EV3. Determination of the DPF-3 cleavage motif.

(**A**) Scatter plot of experimentally determined peptide half-lives upon incubation with DPP4 (from (Keane et al, 2011)) vs. DPF-3 qPISA model substrate score. Each dot is a unique peptide whose first three amino acids are indicated. (**B**) Sequence logo showing the differential cleavage activity (DPP4 subtracted from DPF-3) when the P1 position is occupied by a given amino acid in relation to different amino acids occupying P2 and P1′ position.

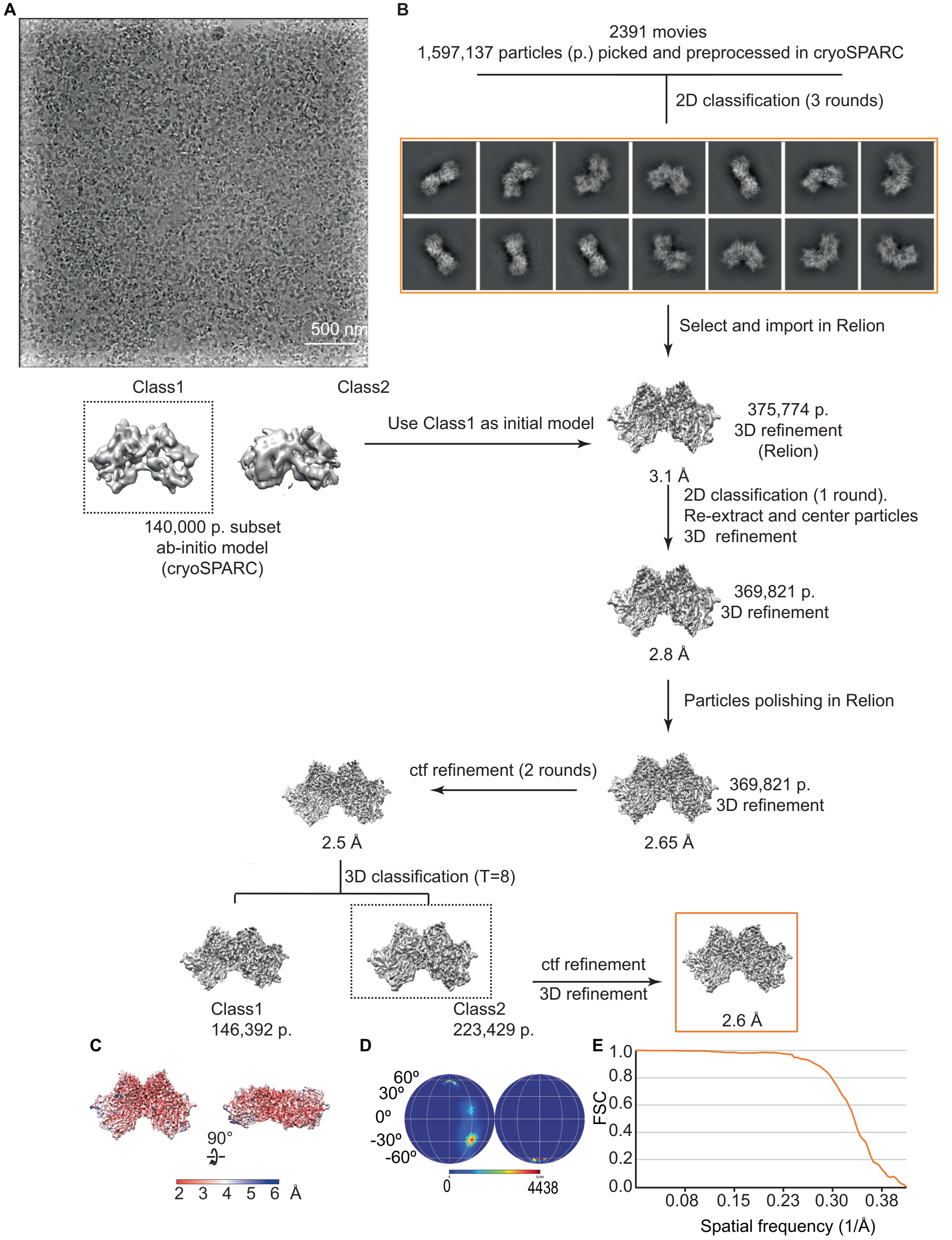
A
500 nm
B
2391 movies
1,597,137 particles (p.) picked and preprocessed in cryoSPARC
2D classification (3 rounds)
Select and import in Relion
Class1
Class2
Use Class1 as initial model
140,000 p. subset
ab-initio model
(cryoSPARC)
375,774 p.
3D refinement
(Relion)
3.1 Å
2D classification (1 round).
Re-extract and center particles
3D refinement
369,821 p.
3D refinement
2.8 Å
Particles polishing in Relion
ctf refinement (2 rounds)
369,821 p.
3D refinement
2.65 Å
2.5 Å
3D classification (T=8)
Class1
146,392 p.
Class2
223,429 p.
ctf refinement
3D refinement
2.6 Å
C
90°
2 3 4 5 6 Å
D
60°
30°
0°
-30°
-60°
0
4438
E
FSC
1.0
0.8
0.6
0.4
0.2
0.0
0.08
0.15
0.23
0.30
0.38
Spatial frequency (1/Å)

 © The Author(s)

◀ **Figure EV4. Classification and refinement workflow for the DPF-3 homodimer complex.**

(**A**) Representative cryo-EM micrographs denoised with Topaz, see methods. (**B**) The movies were imported in cryoSPARC for initial data processing including 2D classification (representative 2D class averages are shown inside an orange frame). The best particles were imported in Relion. Ab initio model generation was performed in cryoSPARC, while 3D classification and refinement were performed in Relion. The final model includes 223,429 particles. The boxes defined by a dashed line indicate the good models and set of particles used for the following step in the data processing workflow. (**C**) Local resolution filtered map (MonoRes). (**D**) Angular distribution for the particles leading to the final EM map. (**E**) Gold-standard Fourier shell correlation curve.

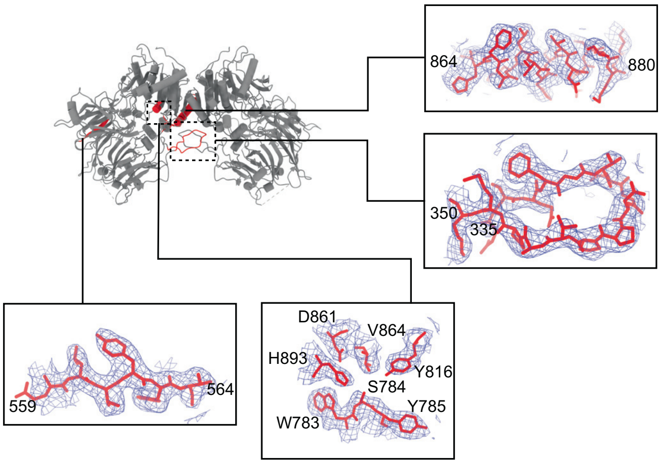

Figure EV5. Model map of DPF-3 dimer.

Representative model-map overlays (insets) for regions colored in red in the top left panel. Numbers correspond to sequence positions of residues. The map was sharpened with LocScale and the contour level is 0.192.

© The Author(s)
